# Post-reperfusion acute MR diffusion in stroke is a potential predictor for clinical outcome in rats

**DOI:** 10.1038/s41598-023-32679-1

**Published:** 2023-04-05

**Authors:** Szilvia Anett Nagy, Ivan Ivic, Péter Tóth, Sámuel Komoly, Tamás Kiss, Máté Pénzes, András Málnási-Csizmadia, Tamás Dóczi, Gábor Perlaki, Gergely Orsi

**Affiliations:** 1 ELKH-PTE Clinical Neuroscience MR Research Group, Ret Str. 2, 7623 Pecs, Hungary; 2 Pecs Diagnostic Centre, Rét Street 2, 7623 Pecs, Hungary; 3 Structural Neurobiology Research Group, Szentágothai Research Centre, University of Pecs, Ifjúság Street 20, 7624 Pecs, Hungary; 4 Department of Neurology, Medical School, University of Pecs, Rét Street 2, 7623 Pecs, Hungary; 5 Selvita d.o.o., Prilaz Baruna Filipovića 29, 10000 Zagreb, Croatia; 6 Department of Neurosurgery, Medical School, University of Pecs, Rét Street 2, 7623 Pecs, Hungary; 7 Szentágothai Research Centre, University of Pecs, Ifjúság Street 20, Pecs, Hungary; 8 Department of Biochemistry, Eötvös Loránd University, Pázmány Péter Sétány 1/C, 1117 Budapest, Hungary; 9 Motorpharma Ltd., Szilágyi E. Fasor 27, 1026 Budapest, Hungary; 10 ELKH-ELTE Motor Pharmacology Research Group, Department of Biochemistry, Eötvös Loránd University, Pázmány Péter Sétány 1/C, 1117 Budapest, Hungary

**Keywords:** Experimental models of disease, Neuroscience, Stroke

## Abstract

Middle cerebral artery occlusion (MCAO) models show substantial variability in outcome, introducing uncertainties in the evaluation of treatment effects. Early outcome predictors would be essential for prognostic purposes and variability control. We aimed to compare apparent diffusion coefficient (ADC) MRI data obtained during MCAO and shortly after reperfusion for their potentials in acute-phase outcome prediction. Fifty-nine male rats underwent a 45-min MCAO. Outcome was defined in three ways: 21-day survival; 24 h midline-shift and neurological scores. Animals were divided into two groups: rats surviving 21 days after MCAO (survival group, n = 46) and rats dying prematurely (non-survival/NS group, n = 13). At reperfusion, NS group showed considerably larger lesion volume and lower mean ADC of the initial lesion site (p < 0.0001), while during occlusion there were no significant group differences. At reperfusion, each survival animal showed decreased lesion volume and increased mean ADC of the initial lesion site compared to those during occlusion (p < 10^–6^), while NS group showed a mixed pattern. At reperfusion, lesion volume and mean ADC of the initial lesion site were significantly associated with 24 h midline-shift and neurological scores. Diffusion MRI performed soon after reperfusion has a great impact in early-phase outcome prediction, and it works better than the measurement during occlusion.

## Introduction

Stroke is one of the most common causes of disability and the second leading cause of death worldwide. The stroke-related annual cost was estimated at $36.5 billion in the US in 2010 and €60 billion in 32 European Countries in 2017^1–3^. Between 2017 and 2047, the absolute burden of stroke is expected to continue in the European Union, particularly in Eastern states^4^.

Middle cerebral artery (MCA) is the most common site of human ischemic stroke^5^, thus MCA occlusion (MCAO) has emerged as the most prevalent animal model of focal cerebral ischemia^6^. However, MCAO is often associated with poor functional outcome and high mortality, which—in combination with high outcome variability—are considered to be the main reasons for the translational failure of effective preclinical stroke treatments^7^. Predicting outcome in the acute phase of ischemic stroke (within the first few hours) may allow researchers to stratify animals relying on the expected outcome and control variability-related bias in neuroprotection studies^8^. However, only few approaches are used to quantitatively estimate prognosis, and none of them are standards and widely accepted. For example, early phase behavioral testing (performed during occlusion or within the first 3 h after reperfusion) cannot reliably predict the occurrence and magnitude of cerebral infarct or identify successfully operated rats^9,10^, the acute neuro-score grade does not necessarily correspond to animal survival or death^9^ and the neurological scores recorded during occlusion show no correlation with the same scores assessed 24 h post-reperfusion^10^. However, 23–24 h postreperfusion timepoint was shown to be already late enough to be reliable for behavioral/neurological testing both in mice and rats^10,11^. Another possibility is Laser Doppler Flowmetry (LDF), which is a widely used approach to confirm proper MCA occlusion in experimental studies^12,13^. However, this method is relatively invasive compared to MRI^14^ and single-site LDF monitoring of lateral MCA territory—which is most commonly used to confirm successful MCAO—is not predictive for 24 h ischemic outcome, neither in terms of infarct volume nor neurological score^8^.

Magnetic resonance imaging (MRI) is nowadays also widely available in experimental research and various techniques are used for stroke outcome prediction^15,16^. Among these techniques, diffusion MRI has become the mainstay of acute stroke imaging, probably because of its capability to demonstrate acute infarction within minutes of symptom onset^16–18^. Nevertheless, acute diffusion MRI acquired during occlusion or soon after reperfusion was shown to have only limited ability to assess final lesion size (i.e. anatomical outcome)^15^. Even if diffusion maps of these two timepoints are combined, the prediction looses accuracy in half of the test group animals (i.e. 3 out of the 6 cases)^15^. Using a murine model it was demonstrated that the accuracy of infarct volume prediction by early MRI highly depends on MCAO duration and the time of imaging^19^. Post-reperfusion apparent diffusion coefficient (ADC) shows a biphasic behavior characterized by an acute normalization followed by a secondary decline^20^ and importantly this behavior resembles that observed in humans after reperfusion induced by thrombolytic therapy^21,22^. However, the normalization and secondary decline of ADC do not seem to be associated with neurological recovery and worsening, respectively^23^ and reperfusion-induced acute normalization of diffusion MRI abnormality is known not to definitely reflect tissue salvage or the reversal of neuronal shrinkage and astrocytic swelling induced by stroke^20,21^.

Acknowledging the limitations of early phase diffusion MRI as a predictor of final anatomical outcome and keeping in mind that MRI detectable changes in ischemia-damaged rat brain continue for at least 1 year post-ischemia^24^, instead of predicting “final lesion size”, we aimed to assess the potential of acute diffusion MRI in predicting more direct clinical outcome variables. More specifically, acute diffusion MRI data acquired during occlusion and early after reperfusion were investigated for their potential in predicting survival or death, midline-shift and neurological deficits following transient MCAO in rat.

## Methods

### Animal preparation

Eighty-eight young male *Wistar* SPF rats (Toxi-Coop Ltd., Balatonfüred, Hungary) aged 6–7 weeks (150–175 g) upon arrival, were group-housed in plastic cages (378 mm × 217 mm × 180 mm, equipped with feeder and bottle container) under standard animal room conditions (temperature 22 ± 2 °C; humidity 45–65%; 12 h light/dark cycle; milled chow and water ad libitum). Animals were anesthetized during the surgical and MRI procedures. Briefly, anesthesia was induced with 3–5% isoflurane in 1:2 mixture of O_2_/N_2_O and maintained with isoflurane reduced to 1.85–2.5%. Animal heating was employed for both surgical procedure and MR imaging to maintain the body temperature at 37 °C. Respiration rate, along with rectal temperature was monitored during the experiments.

### Experimental design

**Figure 1:**
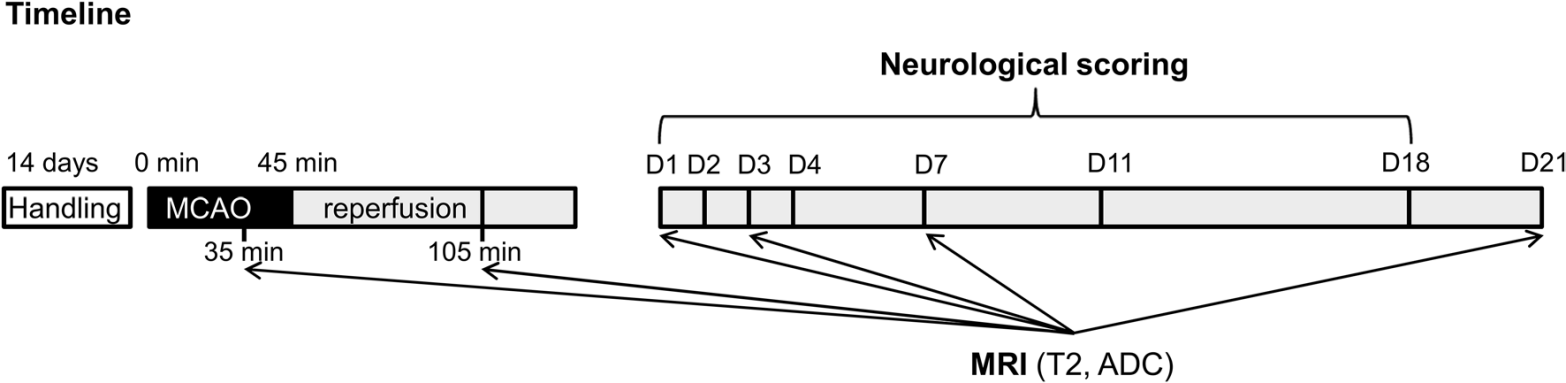
Schematic representation of experimental protocol. *MCAO* middle cerebral artery occlusion, *D1-21* Day 1–21, *T2* T2-weighted imaging, *ADC* apparent diffusion coefficient measurement; black color represents the 45-min period of filament occlusion; gray indicates the 21 day period after reperfusion.

The full experimental design and the timeline of the procedures are depicted on Fig. 1. First, the animals were allowed to habituate to the new housing conditions for 2 weeks. During this habituation period and throughout the entire experiment all animals were handled daily. After the 2-week habituation period—when animals were 8–9 week old young adults—rats underwent a transient MCAO procedure lasting 45 min. Animals that died during surgery or before reperfusion MRI (n = 11), those with unsuccessful MCAO-induced ischemia (i.e. without any lesion or with only subcortical lesion appearance during the occlusion as visually determined based on the ADC map; n = 12) or those with hemorrhagic transformation during the follow-up (n = 4) were excluded from the study. Two animals were excluded due to insufficient imaging quality. Exclusion criteria were established prior to data analysis and single animal represents the experimental unit. The remaining 59 animals were included in the final evaluation. The initial body-weight was measured right before the surgery.

The study endpoint was 21 days after MCAO, but based on predefined ethical endpoints, animals being in a very poor condition (n = 3)—comatose state; tetraplegia; any other condition preventing the animal from taking food and/or fluids for a considerable period of time—were euthanized earlier by intraperitoneal application of sodium pentothal (200 mg/kg body weight).

Diffusion MRI was performed: (i) ~ 35 min after filament insertion (i.e. during occlusion; mean ± SD: 35:04 ± 02:05; range: 30:31–39:08 mm:ss after filament insertion), (ii) ~ 1 h after reperfusion (i.e. reperfusion measurement; mean ± SD: 01:02:59 ± 00:07:36; range: 00:54:08–01:50:21 hh:mm:ss after reperfusion) and (iii) Day 1, 3, 7 and 21 after MCAO procedure (i.e. D1, D3, D7 and D21). Animals were continuously anaesthetized from the start of surgery until the end of the reperfusion measurement.

Neurological testing was performed on Day 1–4, 7, 11 and 18 after MCAO.

Keeping in mind that the development of significant vasogenic edema may pseudonormalize diffusion coefficients and the fact that our goal was to examine the prognostic ability of hyperacute (0–6 h) MRI, only diffusion measurements obtained at the hyperacute phase (i.e. during occlusion and one hour after reperfusion) were considered for evaluation. Midline-shift (MLS) as an outcome indicator was assessed from T2 measurement on Day 1, when MLS was demonstrated to reflect the amount of vasogenic brain edema^25^.

### MCAO procedure

Animals were subjected to transient Koizumi-type MCAO as described earlier^26^. Following the midline incision of the neck, the left common carotid artery (CCA), left external carotid artery (ECA), and left internal carotid artery (ICA) were identified and isolated from the surrounding tissues. CCA and ECA were ligated using silk suture (Silk suture USP 1, KRUUSE, Langeskov, Denmark) while ICA was clipped with clamp (FST, No. 18055-04). CCA incision was made and a silicone rubber-coated monofilament^7^ (503345PK5Re, 403545PK10Re, 403745PK10Re, or 403945PK10Re, Doccol Corporation) was inserted, gently ligated with a silk suture to allow insertion of filament and preventing blood loss. Filament size was chosen based on animal weight and manufacturer’s recommendations. The clamp was removed, and the filament was pushed cranially until a mild resistance was felt indicating the occlusion of the left middle cerebral artery, approximately 20 mm from CCA bifurcation. For reperfusion, the filament was carefully removed after 45 min, and ligation of ICA was performed. The wound was disinfected with 70% ethanol and closed.

### Neurological scoring tests

To assess the impact of ischemia and reperfusion on locomotor movement, neurological tests were adopted from the scoring system of Clark et al. originally described for mice^27^. Due to the invasive nature of MCAO, the first neurological scoring tests were carried out 24 h after the procedure. The neurological status of the animals was assessed by the same person—blinded to MRI data analyses results—on Day 1–4, 7 and 18 after the MCAO. A slight modification in the scoring system was implemented as climbing was not tested.

Focal deficit score was defined as the sum of the following items rated between 0 and 4 depending on the severity: (1) body symmetry, (2) gait, (3) circling behaviour, (4) front limb symmetry, (5) compulsory circling and (6) whisker-response.

General deficit score was defined as the sum of the following items: (1) hair: rated between 0 and 2, (2) ears: rated between 0 and 2, (3) eyes: rated between 0 and 4, (4) posture: rated between 0 and 4, (5) spontaneous activity: rated between 0 and 4, (6) epileptic behavior: rated between 0 and 12 in the following discrete steps: 0, 3, 6, 9 and 12.

Total neurological score was calculated as the sum of focal (rated between 0 and 24) and general deficit scores (rated between 0 and 28). The maximum total neurological score an animal can reach is 52 points.

### Magnetic resonance imaging

Imaging was performed on a 4.7 T small-animal MRI system (Pharmascan 47/16 US; Bruker BioSpin MRI GmbH, Ettlingen, Germany) running Paravision 6.0.1 and equipped with B-GA9S HP gradient system (380 mT/m gradient strength and 3420 T/m/s maximum slew rate). Images were acquired with a transmit-only volume coil (outer/inner diameter = 89/72 mm) and a receive-only rat brain surface coil.

Coronal T2-weighted images were obtained using a 2D RARE sequence: TR/TE = 3470/36 ms; 30 slices; slice thickness = 0.7 mm; interslice gap = 0.3 mm; field of view (FOV) = 35 × 35 mm^2^; matrix = 256 × 256; echo spacing = 12 ms; rare factor = 8; fat suppression = on; averages = 2; bandwidth = 128 Hz/pixel; acquisition time = 3:42.

To map ADC, a double-sampled respiratory-triggered segmented spin-echo echo-planar imaging sequence with diffusion-weighting gradients applied in 3 orthogonal directions was used: TR/TE = 2800/30 ms; 19 coronal slices; slice thickness = 1 mm; interslice gap = 0.25 mm; FOV = 30 × 30 mm^2^; matrix = 128 × 128; fat suppression = on; segments = 3; averages = 6; b-values = 0, 800, 1500 s/mm^2^; bandwidth = 2344 Hz/pixel; field map-based shimming (MAPSHIM) using an elliptical shim volume adjusted to the rat brain; acquisition time: 10:12 ± 00:47 (mm:ss) depending on respiratory rate. ADC maps were automatically calculated by the post-processing macro of Paravision software.

### MRI data analysis

Images were first converted from Bruker to NifTI format and scaled up by a factor of 10 in each dimension to ensure compatibility with algorithms designed for human brain studies. MRI data analyses were performed by a single observer for each measure, blinded to outcome variables.

### Diffusion MRI

Diffusion images were processed by FSL (http://www.fmrib.ox.uk/fsl, Oxford, UK) tools. Ischemic lesion volumes during the occlusion and at reperfusion were determined based on ADC maps thresholded at a previously published value (5.3 × 10^–4^ mm^2^/s)^28^ followed by manual correction using FSLview to exclude extracerebral regions or other non-infarct-related low ADC regions.

Diffusion measurement at reperfusion was registered (via b = 0 s/mm^2^ volume) to the measurement during occlusion starting with a 12-dof linear and followed by non-linear registration^29,30^. Applying the inverse of this transformation and nearest neighbor interpolation, the initial ADC lesion mask (defined during occlusion and hereafter referred to as initial lesion site) was propagated onto the diffusion measurement obtained at reperfusion to extract mean ADC values of the same anatomical region at both timepoints. Before applying the propagated masks, manual correction (such as excluding any ventricular or extracerebral voxels) were performed if necessary.

### Midline-shift and final lesion size

3D Slicer software (http://www.slicer.org, Version 4.10.2) was used for the analyses of T2-weighted images. Midline-shift was measured on coronal T2-weighted images at 24 h after MCAO using the validated method by Walberer et al.^25^. In brief, distances from the external border of cortex to the middle of third ventricle were measured for both the ipsilateral (D_i_) and contralateral hemispheres (D_c_) at the level of maximum lateral displacement of the third ventricle. Midline-shift was defined as: MLS = (D_i_ − D_c_)/2.

Final lesion size was measured on coronal T2-weighted images acquired 21 days after MCAO. In brief, the hyperintense regions in the ipsilateral hemisphere were manually delineated on each slice without including ventricles. To compensate for the inhomogeneous sensitivity profile of the receive-only rat brain surface coil, all images were bias-corrected with N4ITK algorithm^31^. Raw and bias-field corrected images were viewed simultaneously to facilitate the accurate lesion segmentation.

### Statistical analyses

Statistical analyses were performed using IBM SPSS Statistics for Windows, Version 23.0 (IBM Corp., Armonk, NY, USA). Because some variables were not normally distributed according to Shapiro–Wilk test (e.g. general deficit, lesion volume at reperfusion), subsequent statistical analyses were performed by non-parametric tests.

Animals were divided into two groups defined as (i) rats surviving 21 days after MCAO (i.e. survival group, n = 46) and (ii) rats died within 21 days after MCAO (i.e. non-survival group, n = 13). Because of the post-hoc categorization of animals based on survival, no randomisation or the control of confounders such as the order of measurements or animal cage/location were possible in this design.

ADC lesion volumes and mean ADC of the initial lesion site during occlusion and at reperfusion were compared between the two groups by Mann–Whitney U-tests. Initial body-weight was also compared by Mann–Whitney U-test between the non-survival and survival groups.

Wilcoxon signed-rank test was run separately for both groups to assess diffusion parameter changes between the measurements during occlusion and at reperfusion. For the survival group, lesion size changes from the occlusion as well as from the reperfusion to the final 21 day measurement were also assessed by Wilcoxon signed-rank test.

Midline-shift was compared between non-survival (n = 5) and survival groups (n = 46) by Mann–Whitney U-test. The association of early diffusion measures (ADC lesion volume and mean ADC of the initial lesion site during occlusion and at reperfusion) and MLS were assessed by Spearman’s rank correlation using all available data (n = 51).

Day 1 neurological scores were compared between non-survival (n = 8) and survival groups (n = 46) by Mann–Whitney U-test. The association of early diffusion measures and neurological scores were assessed by Spearman’s rank correlations using all available data (n = 54 at Day 1, n = 48 at Day 2–4 and n = 47 at Day 7–18).

The abilities of diffusion measures during occlusion and at reperfusion to differentiate between survival and non-survival animals were assessed by fitting receiver operating characteristic (ROC) curves and calculating sensitivities, specificities and areas under the ROC curves (AUCs). The statistical comparison among the ROC curves was also performed by DeLong test (MedCalc Statistical Software Ltd, Ostend, Belgium; https://www.medcalc.org)^32^. As a rule of thumb, the following criteria were defined: 0.5 < AUC < 0.7 poor discrimination, 0.7 ≤ AUC < 0.8 acceptable discrimination, 0.8 ≤ AUC < 0.9 excellent discrimination, and AUC ≥ 0.9 outstanding discrimination. Reclassification tables and net reclassification indices were also obtained based on the optimal ROC-curve derived cutoff values of acute stage diffusion measures (i.e. cutoff points nearest to the upper left corner of the ROC space). Kaplan–Meier curves were used to visualize cumulative survival proportions against time in groups defined by the optimal cutoff values of diffusion measures.

### Ethical approval and consent to participate

The experiments were approved by the Hungarian Ethical Committee on Animal Research according to the Ethical Codex of Animal Experiments (License No. BA/73/00940-5/2020) and reported according to the ARRIVE (Animals in Research: Reporting In Vivo Experiments) guidelines.

### Human and animal ethics

All animal procedures were carried out in accordance with the guidelines of Decree No. 40/2013. (II. 14.) of the Hungarian Government and the EU Directive 2010/63/EU. Throughout the entire experiment, adequate measures were taken to minimize pain or discomfort for the experimental animals.

## Results

A data file, containing all raw values behind statistical analysis is provided as Supplementary Material. Initial body-weight—measured right before the surgery—was not significantly different between non-survival and survival animals (2-sided exact p = 0.797; Table 1).

**Table 1:** Descriptive statistics and between group differences.

Metrics	Group (n)	Median [range]	p-value^a^
Initial body-weight (g)	Non-survival (13)	260 [240–298]	0.797
	Survival (46)	267 [217–289]	
Lesion volume during occlusion (mm^3^)	Non-survival (13)	294 [105–496]	0.475
	Survival (46)	257 [117–323]	
Lesion volume at reperfusion (mm^3^)	Non-survival (13)	223 [12–434]	< 0.0001
	Survival (46)	27 [1–168]	
Lesion volume 21 days after MCAO (mm^3^)	Non-survival	n.a.	n.a.
	Survival (46)	170 [5–313]	
Mean ADC of the initial lesion site during occlusion (10^–4^ mm^2^/s)	Non-survival (13)	4.6 [4.2–4.8]	0.578
	Survival (46)	4.5 [4.3–4.7]	
Mean ADC of the initial lesion site at reperfusion (10^–4^ mm^2^/s)	Non-survival (13)	5.0 [3.9–6.9]	< 0.0001
	Survival (46)	6.6 [4.7–8.1]	
Midline-shift 24 h after MCAO (mm)	Non-survival (5)	0.920 [0.005–1.150]	0.032
	Survival (46)	0.403 [0.000–0.830]	
General deficit 24 h after MCAO	Non-survival (8)	12.5 [9.5–20.5]	0.0014
	Survival (46)	9 [4.5–19]	
Focal deficit 24 h after MCAO	Non-survival (8)	18 [13–21]	0.0110
	Survival (46)	14.5 [8.5–19]	
Total deficit 24 h after MCAO	Non-survival (8)	30.8 [23–34.5]	0.0004
	Survival (46)	24 [15.5–35.5]	

*N* number of animals in each group, *Initial body-weight* body-weight right before left middle cerebral artery occlusion (MCAO) surgery, *ADC* apparent diffusion coefficient, *n.a.* not applicable.

^a^Mann–Whitney U-test (2-sided exact p-value).

### Association between survival and early diffusion measurements

**Figure 2:**
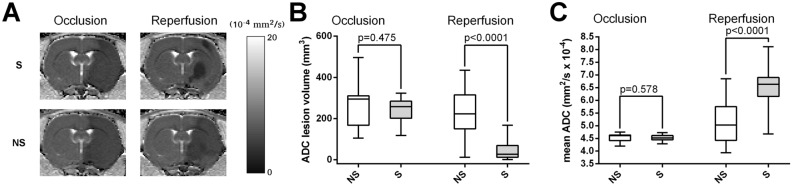
Apparent diffusion coefficient (ADC) maps show larger ischemic stroke volume at reperfusion in rats not surviving ischemia–reperfusion injury. (**A**) Representative ADC maps measured from a survival (S) rat (surviving ischemia–reperfusion injury) and from a non-survival (NS) rat (died at 48 h after reperfusion) during left middle cerebral artery occlusion (MCAO) and at reperfusion. (**B**) Summary data of differences between survival (S) and non-survival (NS) rats (n = 46, n = 13 respectively) in ADC lesion volume (mm^3^) during occlusion of left middle cerebral arteries and at reperfusion. (**C**) Differences between survival (S) and non-survival (NS) rats in mean ADC of the initial lesion site (mm^2^/s × 10^–4^) during occlusion of left middle cerebral arteries and at reperfusion. Whiskers are set at minimum and maximum, the horizontal line marks the median, whereas box indicates the interquartile range (25–75%). 2-sided exact p-values are based on Mann–Whitney U-test.

During occlusion, lesion volume did not show any statistically significant difference between non-survival and survival groups (Fig. 2B, Table 1). However, at reperfusion, lesion volume was considerable larger in the non-survival group (Fig. 2B, Table 1).

Similarly, mean ADC values, calculated from the initial lesion, showed no difference between the two groups during occlusion, while mean ADC from the same anatomical region was significantly lower in the non-survival group at reperfusion (Fig. 2C, Table 1).

**Figure 3:**
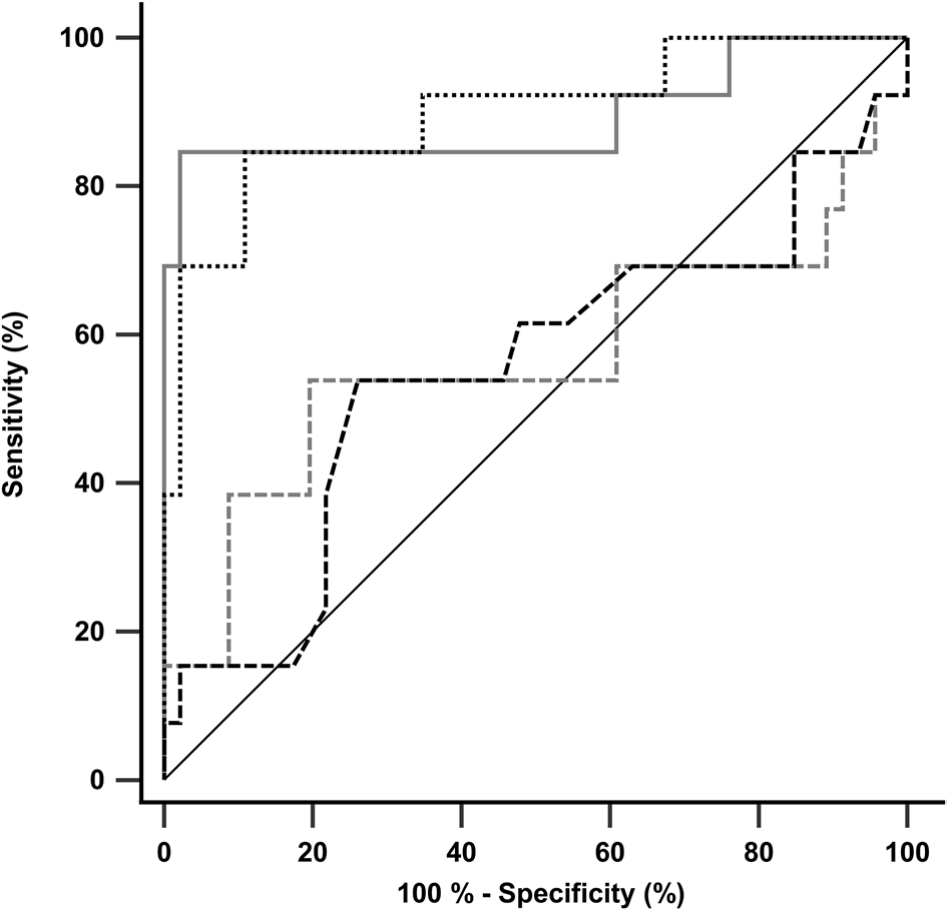
Receiver operating characteristic (ROC) curves of the acute stage diffusion measures for identification of animals dying prematurely. ROC curves of lesion volume during occlusion (dashed gray line), lesion volume at reperfusion (solid gray line), mean ADC of the initial lesion site during occlusion (dashed black line) and mean ADC of the initial lesion site at reperfusion (dotted black line) are shown.

The discriminative performance of acute stage diffusion measures for early identification of premature death was shown by AUC, sensitivity and specificity values for each measure (Table 2). Statistical comparisons of the ROC curves showed significantly higher AUCs for diffusion measures at reperfusion compared to those during occlusion (0.0008 ≤ p-value ≤ 0.0175, Table 2), while diffusion measures at the same timepoint did not differ significantly (p ≥ 0.8571, Table 2). Based on AUCs < 0.7, a poor discrimination between survival and non-survival animals is considered for the diffusion measures during occlusion, while AUCs > 0.89 put the discrimination of the diffusion measures at reperfusion at the upper border of excellent discrimination. ROC curves are shown in Fig. 3. To assess more comprehensively how one diffusion measure works compared to the others, reclassification tables and the corresponding net reclassification indices (NRIs) were added as a Supplementary Table (Supplementary Table 1). Kaplan–Meier curves illustrating the cumulative survival functions for animals grouped according to optimal ROC-derived cutoff points of acute stage diffusion measures are shown in Supplementary Fig. 1.

**Table 2:** ROC curve analysis of the diffusion measures.

Diffusion measure	Optimal cutoff	Sensitivity (%)	Specificity (%)	AUC	Comparison of ROC curves		
					vs. lesion volume_rep_	vs. mean ADC_occl_	vs. mean ADC_rep_
Lesion volume_occl_	> 289.8 mm^3^	53.8	80.4	0.567	**p = 0.0008**	p = 0.9370	**p = 0.0015**
Lesion volume_rep_	> 145.4 mm^3^	84.6	97.8	0.891	–	**p = 0.0175**	p = 0.8571
Mean ADC_occl_	> 4.605 × 10^–4^ mm^2^/s	53.8	73.9	0.552	–	–	**p = 0.0102**
Mean ADC_rep_	< 5.845 × 10^–4^ mm^2^/s	84.6	89.1	0.898	–	–	–

*ROC* receiver operating characteristic, *ADC* apparent diffusion coefficient, *Optimal cutoff* closest to the upper left corner of the ROC space, *AUC* area under the curve, *lesion volume*_*occl./rep.*_ lesion volume determined based on ADC map during occlusion/at reperfusion, *mean ADC*_*occl./rep.*_ mean ADC of the initial lesion site during occlusion/at reperfusion; Sensitivities and specificities are reported at the optimal cutoff values for identification of animals dying prematurely.

p-values were obtained from pairwise comparison of ROC curves by DeLong test. p-values in bold indicates significantly different AUCs.

Each animal in the survival group (n = 46) showed decreased ADC lesion volume at reperfusion compared to that during occlusion, thus Wilcoxon signed-rank test indicated a statistically significant difference between the two timepoints (2-sided exact p < 10^–6^, example shown in Fig. 2A). On the other hand, non-survival group showed more mixed pattern (4/9 rats with increased/decreased lesion volume at reperfusion) resulting in only a nonsignificant trend towards decreased ADC lesion volume at reperfusion (2-sided exact p = 0.080, example shown in Fig. 2A). Similarly, each survival animal showed increased mean ADC of the initial lesion site at reperfusion compared to that during occlusion (2-sided exact p < 10^–6^). The non-survival group also showed increased ADC at reperfusion, but with a less statistically significant p-value (2-sided exact p = 0.017).

Almost each survival animal (i.e. 44 from 46) showed increased final lesion volume compared to that at reperfusion and most of the animals (i.e. 40 from 46) showed decreased final lesion volume compared to that during occlusion, resulting statistically significant differences in both cases (2-sided exact p < 10^–6^).

### Midline-shift

**Figure 4:**
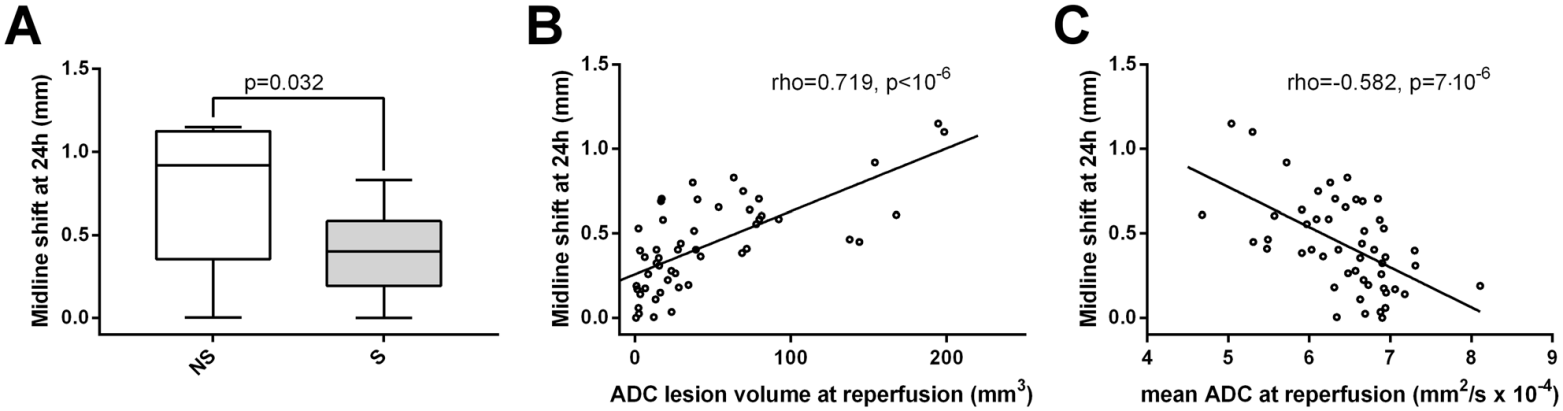
Cerebral midline-shift (assessed 24 h post injury) is increased in rats not surviving ischemia–reperfusion injury, which correlates with lesion volumes calculated by apparent diffusion coefficient (ADC) maps at reperfusion. (**A**) Summary data of differences between survival (S) and non-survival (NS) rats (n = 46, n = 5 respectively) in midline-shift (mm) measured 24 h post-reperfusion. Whiskers are set at minimum and maximum, the horizontal line marks the median, whereas box indicates the interquartile range (25–75%). 2-sided exact p-value is based on Mann–Whitney U-test. (**B**) Correlation between ADC lesion volume (mm^3^) at reperfusion and midline-shift (mm) measured 24 h post-reperfusion in the studied rats (n = 51). rho = Spearman’s rank correlation coefficient. (**C**) Correlation between mean ADC of the initial lesion site at reperfusion (mm^2^/s × 10^–4^) and midline-shift (mm) measured 24 h post-perfusion in the studied animals (n = 51). rho = Spearman’s rank correlation coefficient.

Day 1 midline-shift was significantly higher in the non-survival group (0.78 ± 0.47 vs. 0.40 ± 0.22 mm, 2-sided exact p = 0.032, Fig. 4A; Table 1). ADC lesion volume at reperfusion was positively correlated with midline-shift (Spearman’s rho = 0.719, 2-sided p < 10^–6^, Fig. 4B), while lesion volume during occlusion was not associated with MLS (Spearman’s rho = 0.135, 2-sided p = 0.345). At reperfusion, mean ADC of the initial lesion site was negatively correlated with MLS (Spearman’s rho =  − 0.582, 2-sided p = 7 × 10^–6^, Fig. 4C), while during occlusion, there was a positive association between them (Spearman’s rho = 0.390, p = 0.005).

### Neurological scores

**Figure 5:**
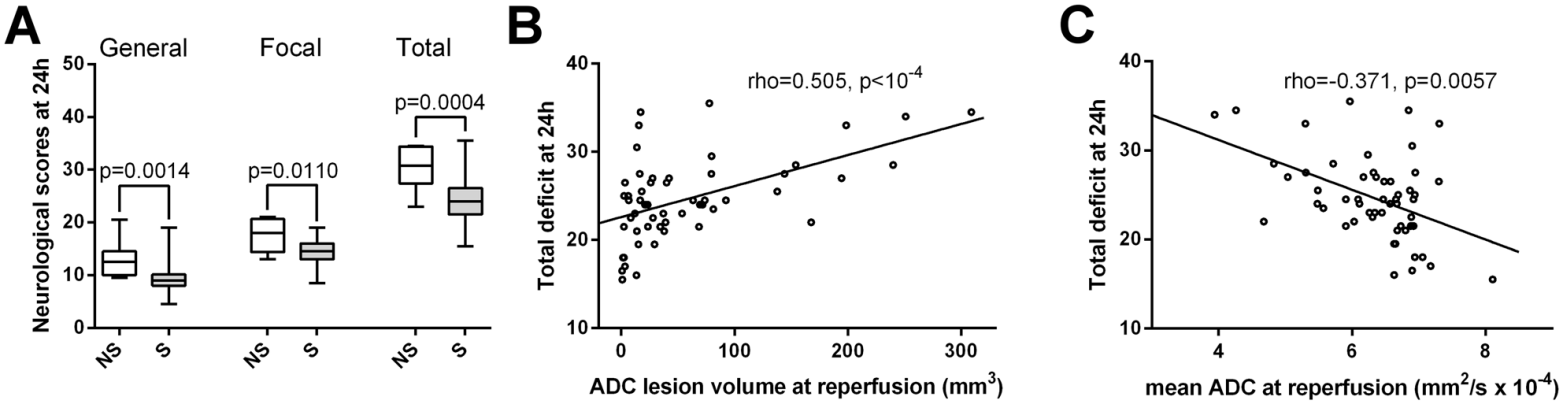
Neurological deficit (assessed 24 h post injury) is increased in rats not surviving ischemia–reperfusion injury, which correlates with lesion volumes calculated by apparent diffusion coefficient (ADC) maps at reperfusion. (**A**) Differences between survival (S) and non-survival (NS) rats (n = 46, n = 8 respectively) in general, focal and total neurological deficit scores assessed at 24 h after reperfusion. Whiskers are set at minimum and maximum, the horizontal line marks the median, whereas box indicates the interquartile range (25–75%). 2-sided exact p-values are based on Mann–Whitney U-test. (**B**) Correlation between ADC lesion volume (mm^3^) at reperfusion and total deficit measured 24 h post-reperfusion in the studied rats (n = 54). rho = Spearman’s rank correlation coefficient. (**C**) Correlation between mean ADC of the initial lesion site at reperfusion (mm^2^/s × 10^–4^) and total deficit measured 24 h post-reperfusion in the studied rats (n = 54). rho = Spearman’s rank correlation coefficient.

Day 1 general, focal and total neurological deficit scores were significantly higher in the non-survival group (2-sided exact, p = 0.0014, p = 0.0110 and p = 0.0004, respectively; Fig. 5A). Day 1 general, focal and total neurological deficit scores were positively correlated with lesion volume at reperfusion (2-sided p = 0.0025, p < 0.0001 and p < 0.0001, respectively, Fig. 5B) and negatively correlated with mean ADC of the initial lesion site at reperfusion (2-sided p = 0.0190, p = 0.0018 and p = 0.0057, respectively, Fig. 5C). The diffusion measures during occlusion were not related to day 1 neurological scores as demonstrated by non-significant Spearman’s correlations between lesion volume during occlusion and Day 1 general, focal and total neurological deficit scores (2-sided p = 0.692, p = 0.238, p = 0.455, respectively) and non-significant correlations between mean ADC of the initial lesion site during occlusion and Day 1 general, focal and total neurological deficit scores (2-sided p = 0.739, p = 0.078 and p = 0.178, respectively). The same correlation pattern was observed for day 2–4 neurological scores, while neurological scores from later timepoints (day 7 onwards) showed no significant association with diffusion measures at reperfusion either.

## Discussion

Acute ischemic stroke is a dynamic process influenced by several unforeseen events^33^. Substantial interindividual variability in outcome and mortality appears to be an inherent problem associated with intraluminal filament MCAO models of preclinical stroke studies^9,34,35^. Predicting ischemic outcome would allow controlling the variability and contribute to the validity of preclinical neuroprotection studies^8^. Thus, early stroke mortality/outcome prediction is highly important and one of the most difficult tasks. The novel findings of the present paper are that acute diffusion MRI taken after reperfusion can be reliably used in early phase outcome prediction, and it has a greater role in outcome prediction than the measurement carried out during occlusion.

### Association between survival and early diffusion measurements

At reperfusion, the non-survival group showed considerably larger ADC lesion volume and lower mean ADC of the initial lesion site compared to the survival group, while during occlusion there were no significant group differences. These results are most probably related to our observation that all survivals showed ADC lesion volume decrease between occlusion and reperfusion (i.e. reversal of ADC lesion), while the non-survival group showed no significant lesion volume change between these two timepoints. The reversal of acute DWI/ADC lesions early after reperfusion, namely that lesion volume decreases after the restoration of blood flow, is well documented in both animal and human studies^20,36–38^. However, it is controversial how often this phenomenon occurs in practice, and whether it has any clinical relevance. Simpkins et al. reported preliminary evidence that the decrease in stroke volume at 2 h after thrombolysis was associated with clinical improvement and that this early change in stroke volume was even a better marker for response to therapy than the final infarct volume (defined at 24 h) or early reperfusion^36^. Similarly, others reported that diffusion lesion reversal just after finishing thrombolysis is significantly associated with dramatic improvement in the total NIH Stroke Scale score, increased rate of early recanalization, and lower initial glucose level on admission^39^. Some investigators found that early diffusion reversal may occur in 32–86% of stroke patients^22,38,40^, while others concluded that it is uncommon, rarely alters perfusion–diffusion mismatch, and unlikely to be clinically relevant^41^. At least part of the above discrepancies may be attributed to differences in the time interval between stroke onset and recanalization^42^, different ADC fluctuation profiles of different infarct types/lesion territories (i.e. territorial vs. watershed infarction and white matter vs. basal ganglia vs. cortical lesions)^37,41,43^ and different timing of follow-up diffusion scans^38^. Moreover, other methodological factors may also explain the discrepancies, including the applied MRI protocols and exact definition of diffusion lesion reversal (e.g. applying threshold for the volume of reversed lesion; visually verifying the diffusion reversal; manual trace or threshold-based lesion delineation)^38,39^.

In the survival group, our findings showed increased final lesion volume compared to that at reperfusion, indicating that the reversal of ADC lesion early after reperfusion may be a transient phenomenon. While most of the studies agree that reperfusion-induced reversal of ADC lesion is usually a transient phenomenon and a sustained reversal is infrequent^20,36,38–41^, it is much more questionable whether secondary ADC decline (i.e. when restored ADC decreases again hours after reperfusion) is already induced by mechanisms during occlusion or it’s more related to a true secondary injury caused only after reperfusion^37^. Li et al. found that reperfusion-triggered ADC normalization in rats is not associated with the reversal of astrocytic swelling and neuronal shrinkage that initially occur during ischemia^20^. Similarly, Ringer et al. also reported that despite DWI lesion recovery during early reperfusion, the reversed tissue already showed signs of cellular stress and irreversible damage^21^, suggesting that reperfusion-induced ADC normalization does not necessarily indicate normal tissue and secondary ADC decline may be rather related to a slow continuation of initial ischemic changes rather than to a true secondary event^20^. However, studies correlating histological results with MRI should be always viewed with caution due to methodological concerns, including “Some time difference is inevitably present between MRI and histology.”, “How to ensure that the same brain tissue is being compared between MRI and histology?” and “How much of the neuronal damage could be due to tissue fixation for histology?”^44^.

Although acute normalization of ADC during early reperfusion may not definitely reflect tissue salvage^21^, both ADC lesion volume and mean ADC at reperfusion seem to be powerful predictors of clinical outcome. This is demonstrated by high sensitivities and specificities for early identification of premature death and the significantly higher AUCs compared to those of diffusion measures during occlusion. The observed NRI values also suggest that diffusion parameters measured at reperfusion are superior compared to those during occlusion. However, please note that the available number of animals hasn’t made it possible to split the data into training and validation data sets. Therefore, the sensitivity/specificity and NRI values reported at the optimal cutoff values may be biased.

Besides outcome prediction, the transient reversal of ADC lesion early after reperfusion may have further clinical relevance. Early spontaneous recanalization in acute ischemic stroke is a documented phenomenon^45^. Spontaneous recanalization of MCA occlusion in the first 6 h may occur in ~ 19% of patients^46^. Due to the above discussed transient post-reperfusion ADC reversal, if these stroke patients are imaged by acute MRI within a few hours after spontaneous recanalization, the damaged brain region may temporarily become less detectable by diffusion MRI. However, further human studies are needed to verify this speculation.

### Midline-shift

In general, midline-shift is an ominous finding, as it is usually associated with the distortion of brainstem anatomy and the increase of intracranial pressure, both considered as a prevalent sign of poor outcome^47^. The degree of early midline-shift correlates with the likelihood of death following stroke; Pullicino et al. reported that patients with MLS ≥ 4 mm measured on CT within 48 h of stroke onset are at high risk for early death^48^. Lam et al. also concluded that early MLS on the first day is considered a highly specific but insensitive sign of poor outcome^49^. In a rat MCAO experiment, Walberer et al. showed that MLS measured at 24 h correlated with the total amount of brain edema^25^. Our results, assessed at Day 1, are in agreement with the above-listed findings, showing significantly higher MLS in the non-survival group, as compared to the survival animals. ADC lesion volume measured at reperfusion was also significantly associated with MLS measured at Day 1, while ADC lesion volume at occlusion showed no such association. The mean ADC of the initial lesion site at reperfusion showed a significant negative correlation with MLS at Day 1. These findings may indicate that a higher degree of early ADC normalization is associated with a lower extent of MLS and consequently, better outcome/survival.

### Neurological scores

At reperfusion, ADC lesion volume showed positive, and mean ADC of the initial lesion site showed negative correlation with neurological scores recorded at days 1–4 after MCAO, while diffusion measurements during occlusion were not associated with neurological scoring. Since the neurological scoring system used in the present study was previously validated to provide clinically relevant outcome assessment^27^, these correlations provide further support for *diffusion measurements at reperfusion* as a useful early predictor of outcome. Interestingly the correlations with neurological scores disappeared from day 7. However, it should be noted that the applied scoring system was originally validated at 48 h post-ischemia^27^ and—similarly to other simple behavioral/neurological tests—it may be not useful for long-term studies^50^.

### Limitations

Our study is not without limitations. First, this study was conducted using a 45-min transient MCAO filament model in *Wistar* rats. The results should be validated in different strains/species, different stroke models and different occlusion times. Our method is based on diffusion measurements performed early after reperfusion, thus it is inherently incapable of providing prognosis before reperfusion or in case of permanent stroke models. Another limitation is the lack of MR perfusion imaging, thus the predictive value of our method could not be compared with perfusion or combined diffusion-perfusion measurements.

Given the effects of estrogen hormones on brain ischemia^51,52^ and that middle-aged or elderly animals are unlikely to fit into the rat brain surface coil of our MR machine, we only included healthy young male rats, that may limit the generalizability of our findings.

## Conclusions

In conclusion, our data indicate that ADC lesion measured soon after reperfusion is strongly associated with outcome in transient rat MCAO model. To assess outcome in the acute phase, the measurement of ADC lesion shortly after reperfusion is advised over the measurement during occlusion. Homogenizing for ADC lesion volume at reperfusion may allow control of outcome variability of stroke models at a very early stage of the experiment, thereby allowing more controlled evaluation of experimental therapies. Our results may also contribute to the development of new diagnostic tools for quantitatively estimating early prognosis. Future studies are needed to evaluate the use of ADC lesion volume defined at reperfusion as a potential acute outcome predictor in humans.

## Supplementary Information

Supplementary Information 1.

Supplementary Information 2.

## Data Availability

Data that support the findings of this study are available as Supplementary Material.
