# Supplementary material for: Post-reperfusion acute MR diffusion in stroke is a potential predictor for clinical outcome in rats
Source: Sci Rep. 2023 Apr 5;13:5598. doi: 10.1038/s41598-023-32679-1 (PMC10076321; doi:10.1038/s41598-023-32679-1)
Supplement: Supplementary file 2 — Supplementary Information 2. [file 41598_2023_32679_MOESM2_ESM.pdf]

Supplementary Information

Post-reperfusion acute MR diffusion in stroke is a potential predictor for clinical outcome in rats

Szilvia Anett Nagy[1,2,3,4], Ivan Ivic[2,5], Péter Tóth[1,6], Sámuel Komoly[4], Tamás Kiss[7], Máté Pénzes[8,9], András Málnási-Csizmadia[9,10], Tamás Dóczi[2,6], Gábor Perlaki[1,2,4,6*] and Gergely Orsi[1,2,4,6*]

[1]ELKH-PTE Clinical Neuroscience MR Research Group, Hungary

[2]Pecs Diagnostic Centre, Hungary

[3]Structural Neurobiology Research Group, Szentágothai Research Centre, University of Pecs, Hungary

[4]Department of Neurology, Medical School, University of Pecs, Hungary

[5]Selvita d.o.o., Zagreb, Croatia

[6]Department of Neurosurgery, Medical School, University of Pecs, Hungary

[7]Szentágothai Research Centre, University of Pecs, Hungary

[8]Department of Biochemistry, Eötvös Loránd University, Hungary

[9]Motorpharma Ltd., Hungary

[10]ELKH-ELTE Motor Pharmacology Research Group, Department of Biochemistry, Eötvös Loránd University, Hungary

[*]These authors contributed equally to this work.

Corresponding author:
Szilvia Anett Nagy
ELKH-PTE Clinical Neuroscience MR Research Group, Ret str. 2, 7623, Pecs, Hungary.
Tel: +36 (72) 535-900
Email: szilvia.anett.nagy@gmail.com

Supplementary method

A practical guide to better understanding Kaplan-Meier curves is provided by Rich et al. [1]. Briefly, Kaplan-Meier curve shows the cumulative probability of survival against time. The probability is recalculated every time an event occurs (i.e. at least one animal dies) and the curve decreases accordingly, as shown by downward steps. Based on the optimal cutoff value of the acute stage diffusion measure, the animals were divided into two groups. The Kaplan-Meier curves were created for both groups and displayed on the same graph for visual comparison. A separate graph was created for each of our four diffusion measures.

To assess the relative performance of one acute stage diffusion measure compared to the other in predicting survival and non-survival, net reclassification indices (NRIs) were calculated separately for events (i.e. death) and non-events (i.e. survival) [2,3]. The former represents the change in proportion of correct classification of non-survival animals (NRI_{events}), while the latter indicates the amount of correct reclassification among survivals ($NRI_{non\ events}$). Reclassification tables and net reclassification indices were obtained based on the optimal cutoff values of acute stage diffusion measures.

Supplementary Figure

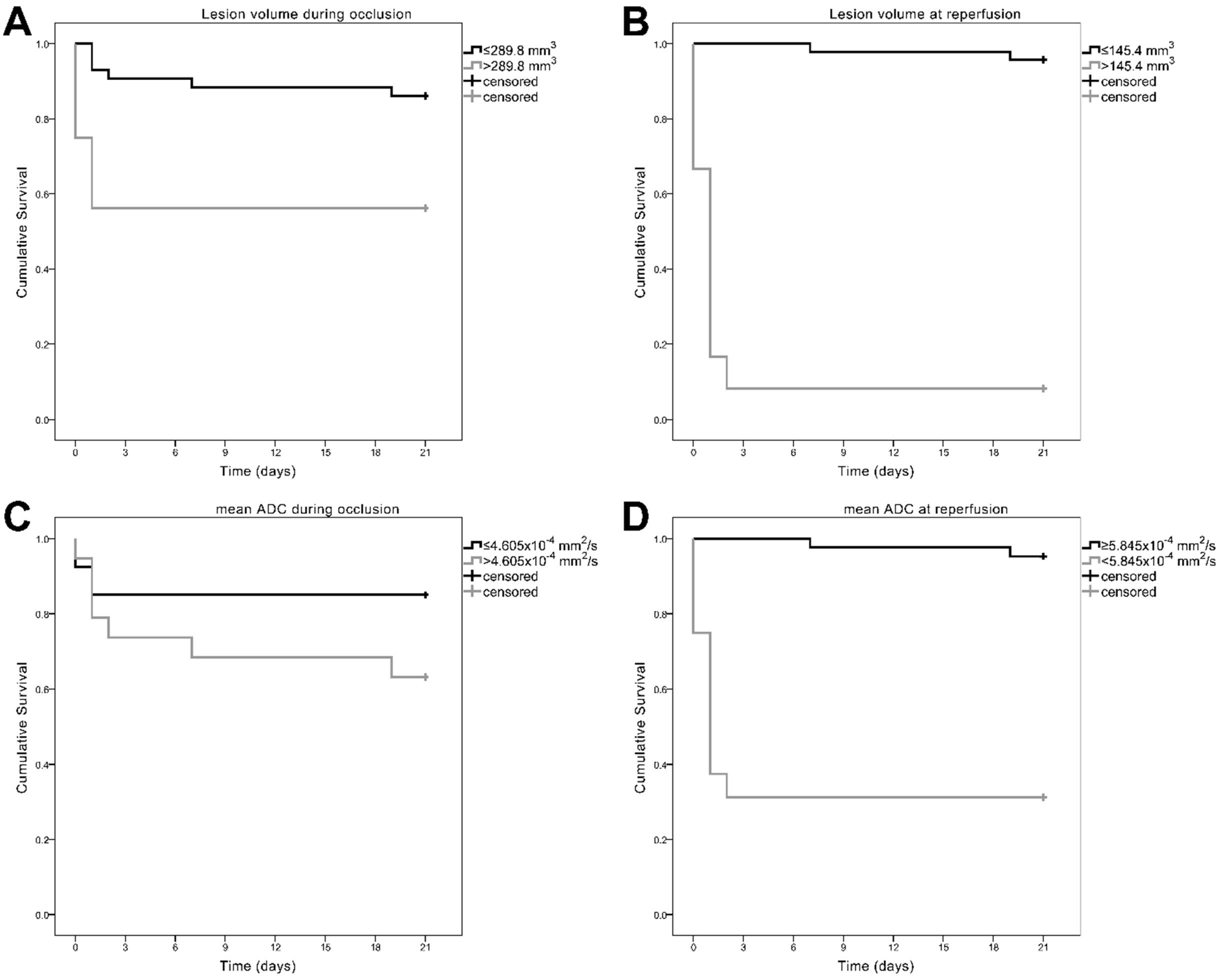

Supplementary Figure 1. Kaplan-Meier curves representing the cumulative survival for the two groups defined according to the optimal cutoff values of lesion volume during occlusion (A), lesion volume at reperfusion (B), mean apparent diffusion coefficient (ADC) of the initial lesion site during occlusion (C) and mean ADC of the initial lesion site at reperfusion (D). The two groups are shown in gray and black on each graph. The x-axis shows the time in days after reperfusion, while y-axis indicates the cumulative probability of surviving a given time.

Supplementary Table

Supplementary Table 1. Reclassification tables for all possible pairings (A-F) of the acute stage diffusion measures

(A)	**lesion volume$_{rep.}$**		
**lesion volume$_{occl.}$**	Survival	Non-survival	
Non-survival animals (n=13)			
Survival	2	**4**	NRI_{events}=0.3077
Non-survival	**0**	7	
Survival animals (n=46)			
Survival	36	**1**	$NRI_{non\ events}$=0.1739
Non-survival	**9**	0	
(B)	**mean ADC$_{occl.}$**		
**lesion volume$_{occl.}$**	Survival	Non-survival	
Non-survival animals (n=13)			
Survival	1	**5**	NRI_{events}=0
Non-survival	**5**	2	
Survival animals (n=46)			
Survival	25	**12**	$NRI_{non\ events}$=-0.0652
Non-survival	**9**	0	
(C)	**mean ADC$_{rep.}$**		
**lesion volume$_{occl.}$**	Survival	Non-survival	
Non-survival animals (n=13)			
Survival	2	**4**	NRI_{events}=0.3077
Non-survival	**0**	7	
Survival animals (n=46)			
Survival	32	**5**	$NRI_{non\ events}$=0.0870
Non-survival	**9**	0	
(D)	**lesion volume$_{rep.}$**		
**mean ADC$_{occl.}$**	Survival	Non-survival	
Non-survival animals (n=13)			
Survival	0	**6**	NRI_{events}=0.3077
Non-survival	**2**	5	
Survival animals (n=46)			
Survival	33	**1**	$NRI_{non\ events}$=0.2391
Non-survival	**12**	0	
(E)	**mean ADC$_{rep.}$**		
**mean ADC$_{occl.}$**	Survival	Non-survival	
Non-survival animals (n=13)			
Survival	0	**6**	NRI_{events}=0.3077
Non-survival	**2**	5	
Survival animals (n=46)			
Survival	29	**5**	$NRI_{non\ events}$=0.1522
Non-survival	**12**	0	
(F)	**lesion volume$_{rep.}$**		
**mean ADC$_{rep.}$**	Survival	Non-survival	
Non-survival animals (n=13)			
Survival	2	**0**	NRI_{events}=0
Non-survival	**0**	11	
Survival animals (n=46)			
Survival	41	**0**	$NRI_{non\ events}$=0.0870
Non-survival	**4**	1	

ADC=Apparent diffusion coefficient; lesion volume$_{occl.}$/$_{rep.}$=lesion volume determined based on ADC map during occlusion/at reperfusion; mean ADC$_{occl.}$/$_{rep.}$=mean ADC of the initial lesion site during occlusion/at reperfusion; NRI_{events}=Net reclassification index for events (i.e. death); $NRI_{non\ events}$=Net reclassification index for non events (i.e. survival). Green (/red) indicates the number of cases correctly (/incorrectly) reclassified with the diffusion measure in the column compared to the diffusion measure in the row. A positive NRI indicates overweight of correctly reclassified cases.

Supplementary references

1 Rich, J. T. *et al.* A practical guide to understanding Kaplan-Meier curves. *Otolaryngol. Head Neck Surg.* **143**, 331-336, doi:10.1016/j.otohns.2010.05.007 (2010).

2 Pencina, M. J., D'Agostino, R. B., Sr., D'Agostino, R. B., Jr. & Vasan, R. S. Evaluating the added predictive ability of a new marker: from area under the ROC curve to reclassification and beyond. *Stat. Med.* **27**, 157-172; discussion 207-112, doi:10.1002/sim.2929 (2008).

3 Kerr, K. F. *et al.* Net reclassification indices for evaluating risk prediction instruments: a critical review. *Epidemiology* **25**, 114-121, doi:10.1097/EDE.0000000000000018 (2014).
